# Comparison of the frequency of viral infections in patients with inborn errors of immunity receiving immunoglobulin by different routes

**DOI:** 10.1007/s00431-025-06201-w

**Published:** 2025-05-30

**Authors:** Hulya Kose, Gozde Ozkan, Abdurrahman Simsek, Yasin Karali, Imran Saglik, Harun Agca, Sara Sebnem Kilic

**Affiliations:** 1 Department of Pediatric Immunology and Rheumatology, Uludag University Faculty of Medicine, Bursa, Turkey; 2 Department of Immunology, Uludag University Faculty of Medicine, Bursa, Turkey; 3 Department of Microbiology, Uludag University Faculty of Medicine, Görükle, Bursa, 16059 Turkey

**Keywords:** PID, CVID, IVIG, CSCIG, FSCIG, Virus, Infection

## Abstract

Immunoglobulin replacement therapy (IRT) is the primary treatment for immunocompromised patients and can be administered via intravenous, subcutaneous, or facilitated subcutaneous routes. In this study, our objective was to compare the incidence of viral infections among patients receiving IRT via different administration routes during the winter season. Fifty-eight patients with primary immunodeficiency (PID) receiving immunoglobulin replacement therapy (IRT) were enrolled in the study. Patients were monitored for their immunoglobulin (Ig) levels, nasal swabs were studied with PCR monthly, and any viral infections were documented. The study included 58 patients with PID, with 33 males (56.8%) and 25 females (43.1%). The median age of the patients was 17 years (IQR, 28.5), and the median age at diagnosis was 11.5 years (IQR, 25.5). The most common IRT route was IVIG, used by 55.1% (*n* = 32) of patients, followed by cSCIG 27.5% (n = 16), and facilitated subcutaneous immunoglobulin (fSCIG) 17% (*n* = 10) of patients. The overall frequency of viral infections was 3.79%, distributed among IRT routes as follows: IVIG (*n* = 32, 4.2%), cSCIG (*n* = 16, 2.5%), and fSCIG (*n* = 10, 4.4%). The infection rate in the cSCIG route was significantly lower than in the IVIG and fSCIG routes (*p* < 0.05). The most common viral agents were adenovirus (21.8%), influenza A (16.4%), and human rhinovirus/enterovirus (16.4%). *Conclusion*: In a 3-month evaluation of patients, the infection rate was lower in the cSCIG route compared to the other IRT routes. cSCIG is a safe and viable treatment option that can effectively improve the quality of life for immunocompromised patients.

**Table:** 

**What Is Known:** *• Immunoglobulin replacement therapy (IRT) is the standard treatment for patients with primary immunodeficiencies (PID), aiming to prevent infections by maintaining adequate serum immunoglobulin levels. Limited research exists regarding the impact of IRT on the prevention of viral infections.* **What Is New:** *• This study reveals key insights into viral infections in PID patients receiving immunoglobulin replacement therapy (IRT) during winter. The overall viral infection rate was 3.79%, with IVIG at 4.2%, cSCIG at 2.5%, and fSCIG at 4.4%. Notably, the cSCIG group had a significantly lower infection rate than the IVIG and fSCIG groups (p0.05). These findings suggest that cSCIG may lower the risk of viral infections in PID patients during high-risk winter months.*

**Supplementary Information:**

The online version contains supplementary material available at 10.1007/s00431-025-06201-w.

## Introduction

Inborn errors of immunity (IEI), encompassing over 450 genetic disorders, increase susceptibility to infections due to deficiencies in specific immune components [1, 2]. Common variable immunodeficiency (CVID) is the most prevalent form of PID, characterized by hypogammaglobulinemia, reduced antibody specificity, and an elevated risk of infections, autoimmunity, and lymphoproliferation. Other common antibody deficiencies include X-linked agammaglobulinemia (XLA), transient hypogammaglobulinemia of infancy, and selective immunoglobulin deficiencies. Immunoglobulin replacement therapy (IRT) is the standard of care for immunodeficiencies that result in impaired antibody production.

Intravenous (IVIG) and subcutaneous (SCIG) administration routes are commonly used, with comparable efficacy [3, 4]. Highly purified human Ig preparations are administered to restore serum IgG levels to physiological concentrations, providing broad-spectrum polyclonal antibodies for immune support. Facilitated subcutaneous immunoglobulin (fSCIG) infusion is a treatment approach that delivers large volumes with minimal needle insertions. Conventional SCIG (cSCIG) therapy typically involves weekly subcutaneous injections to prevent fluctuations in IgG threshold levels [5]. Furthermore, highly concentrated IgG formulations (20%) enable the administration of the required dosage in smaller volumes than less concentrated products. These formulations can be infused quickly and in larger quantities at a single site, offering a more efficient and convenient alternative to traditional subcutaneous preparations [6]. Patients using fSCIG and cSCIG may experience localized side effects, such as urticaria and erythema [5, 6].

The effectiveness and safety of immunoglobulin therapies in controlling infections are critical [7]. A meta-analysis published by Orange et al. [8] demonstrated that IVIG significantly reduced both the frequency and duration of confirmed bacterial lung infections in patients with hypogammaglobulinemia. A study involving 30 patients aged 2 years and older with PID evaluated IVIG, cSCIG, and fSCIG. The study found that the frequencies of severe bacterial infections were similar across all treatment groups throughout the year [9]. While many studies have compared the safety and efficacy of various Ig administration routes, there remains a need for more research on the incidence of viral infections in patients receiving IRT via intravenous, subcutaneous, and facilitated subcutaneous routes [10–12]. In this study, we aimed to compare the incidence of viral infections in immunocompromised patients who received IRT via different routes during the winter season.

## Methods

### Study design

The study used a cross-sectional design from January 1, 2023, to March 31, 2023. The Clinical Research and Ethics Committee of Uludağ University approved the study protocol under ethics approval number 2022–1/22. Informed consent forms were obtained from each participant and their parent/guardian using the Declaration of Helsinki. The study included 58 patients with IEI who were followed up at the Uludağ University Faculty of Medicine, Department of Pediatric Immunology and Rheumatology, and had been receiving Ig replacement therapy for an extended period (> 5 years). From January to March 2023, these patients’ Ig levels were monitored, nasal swabs were collected monthly, and any viral infections were documented when symptoms such as a runny nose, cough, or fever were present. The study was conducted during peak viral infection months to improve the cost-effectiveness of PCR testing. Patients were instructed to report any symptoms of viral infections occurring between monthly PCR analyses. During the study, all patients were contacted by phone, examined when they had symptoms, regardless of their treatment method, and came to take a swab sample when symptoms occurred. Nasal swabs were studied using the PCR method. Serum IgG, IgM, and IgA levels were measured using nephelometry. Patients were randomly selected and divided into three groups based on their current treatment routes: IVIG, cSCIG (10% and 20%), and fSCIG. Personal and family data, clinical findings, immunological test results, and the number of viral and other infections were recorded. Bronchiectasis was diagnosed based on clinical symptoms and chest CT findings. Serum immunoglobulin levels were assessed using endpoint nephelometry.

### Statistical methods

The data was analyzed using the Statistical Package for the Social Sciences (SPSS) 28.0 software. The results were presented as frequencies, percentages, means, standard deviations (SD), median, and interquartile range (IQR). Normality distribution was analyzed using the Kolmogorov–Smirnov (KS) test, and homogeneity of variances was assessed using Levene’s test. Friedman test and generalized linear model (GLM) repeated measures analyses were employed for within-group comparisons of laboratory measurements by month. The Chi-square test was applied to compare infection status based on gender, intravenous immunoglobulin (IVIG) treatment route, and comorbidities. Mann–Whitney test and independent sample test were used to compare infection status with ANC (absolute neutrophil count), ALC (absolute lymphocyte count), immunoglobulin G (IgG), immunoglobulin A (IgA), and immunoglobulin M (IgM) levels. A *p*-value of < 0.05 was considered statistically significant. Paired categorical outcomes were assessed using the McNemar test, while paired continuous variables were evaluated using the Wilcoxon signed-rank test.

## Results

### Participants and descriptive data

The study included 58 patients with IEI, with 33 males (56.8%) and 25 females (43.1%). The median age of the patients was 17 years (IQR, 28.5), and the median age at diagnosis was 11.5 years (IQR, 25.5). The median duration of treatment was 5 years (IQR, 9). The most common IRT route was IVIG, used by 55.1% (*n* = 32) of patients, followed by cSCIG, used by 27.5% (*n* = 16) of patients. All patients received prophylactic antibiotics during the winter months. Among them, 82% were administered trimethoprim/sulfamethoxazole, one dose per day (5 mg/kg), while 8% of patients received azithromycin once daily (5 mg/kg). All IVIG treatments were administered in the hospital, while cSCIG and fSCIG were administered at home. Regarding comorbidities, 14.8% (*n* = 9) of the patients had autoimmune diseases. The distribution of sociodemographic characteristics and clinical and chest CT findings is summarized in Table 1.

**Table 1:** Sociodemographic characteristics, clinical and chest CT findings of patients

Characteristics		Median/*n*	IQR/%
Age		17	28,5
Age at diagnosis		11.5	25.5
Treatment duration		5	9
Gender	Male	33	56.9
	Female	25	43.1
Consanguinity	Yes	19	31.1
	No	42	68.9
IRT route	IVIG	32	55.1
	cSCIG	16	27.5
	fSCIG	10	17.4
Comorbidity	Autoimmune disease	9	14.8
	Asthma	5	8.2
	Malignancy	4	6.6
	COPD	1	1.6
Chest CT findings	Bronchiectasis	6	9.8
	Nodule	4	6.6
	Atelectasis	3	4.9
	Fibrosis	1	1.6
Immunosuppressive therapy		3	4.9

*IQR* interquartile range, *COPD* chronic obstructive pulmonary disease

**Fig. 1:**
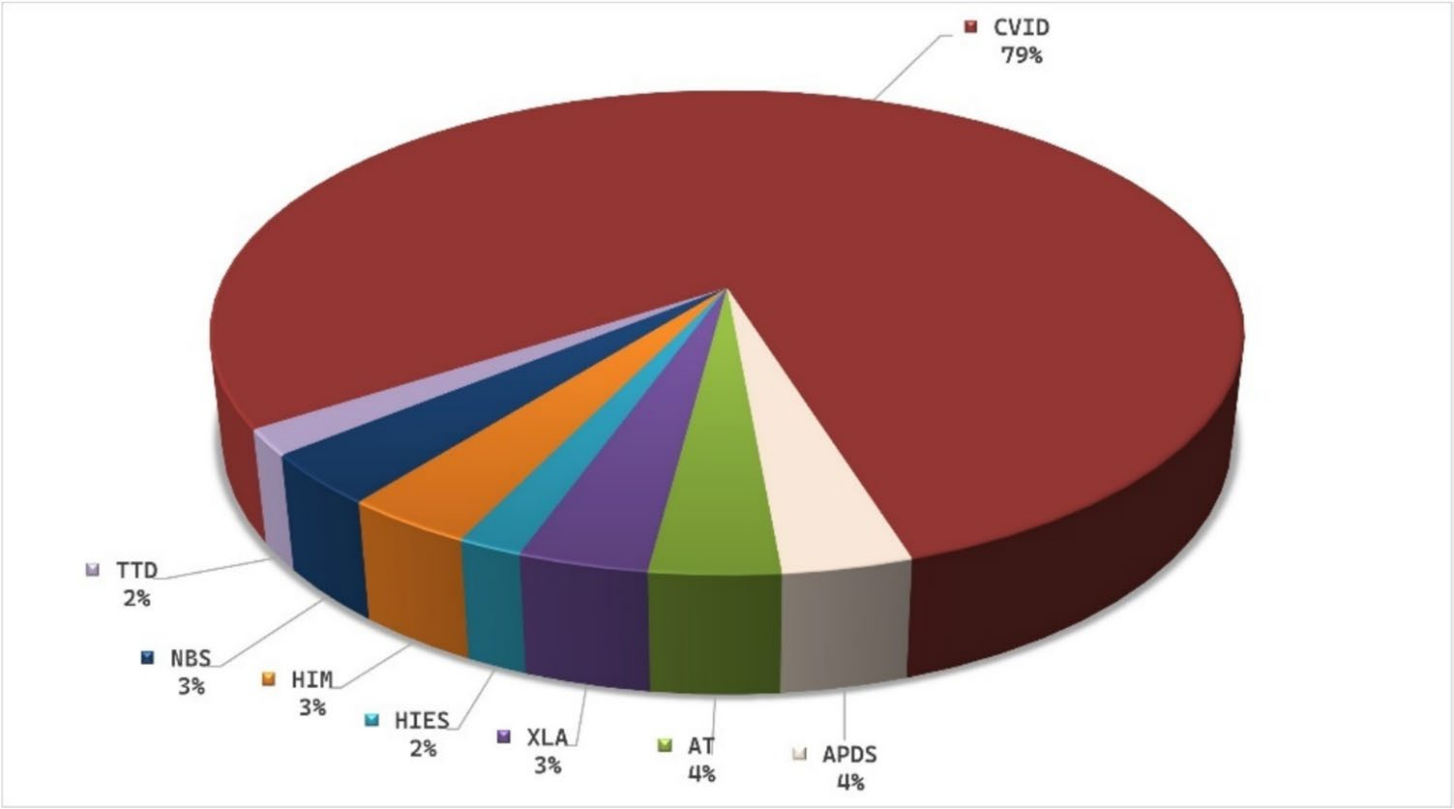
Diagnostic distribution of immunocompromised patients. Abbreviations: CVID, common variable immunodeficiency; APDS, activated PI3 K-delta syndrome; XLA, X-linked agammaglobulinemia;, HIES, hyper IgE syndrome; NBS, Nijmegen breakage sSyndrome; TTD, trichothiodystrophy

The frequency of patients’ diagnoses is shown in Fig. 1, with common variable immunodeficiency (CVID) being the most prevalent (79%). The distribution of other diagnostic groups includes 3% XLA, 4% APDS, 3% NBS, 2% HIES, 3% HIM, 2% TTD, and 4% AT.

In this study, viral infection rates were calculated by analyzing PCR results for each specified infection in every patient and the number of infections over 3 months. Over the 3 months, the overall viral infection frequency was 3.79% (55 infections out of 1450 evaluations). The rates were 7.75% (27 infections out of 348 assessments) in the first month, 3.23% (15 infections out of 464 evaluations) in the second month, and 2.03% (13 infections out of 638 assessments) in the third month. In the 3-month infection rate analysis for the IRT routes, the IVIG route had an infection rate of 4.2% (34 infections in 800 evaluations), the SCIG route 2.5% (10 infections in 400 assessments), and the fSCIG route 4.4% (11 infections in 250 evaluations). Among the Ig treatment groups, the fewest viral infections were observed in those treated with cSCIG (*p* < 0.05). In patients using IVIG and fSCIG, infections were generally detected in the third and fourth weeks. The weekly rates of infections for IRT routes are presented in Supp Fig. 1. During the study, 55.3% of patients experienced at least one infection, while 44.7% did not have any infections over the 3 months. Multiple infection rates were 5% in XLA, 2% in APDS, and 0.49% in CVID patients. There is no statistically significant difference in multiple infections across IRT routes.

The most common viral agents were adenovirus (21.8%), influenza A (16.4%), and human rhinovirus/enterovirus (16.4%). Three-month PCR analysis revealed that patients with X-linked agammaglobulinemia (XLA) (*n* = 4, 6.06%) and those with CVID (*n* = 42, 3.65%) had the highest rates of viral infections (Table 2).

**Table 2:** Distribution of viral agents according to patients’ diagnosis

	CVID *n *= 46	APDS *n* = 2	AT *n* = 2	XLA *n* = 2	HIES *n* = 1	HIM *n *= 2	NBS *n *= 2	TTD *n* = 1	Total infection (3-mo)	Percentage of all infections (%)
Adenovirus	10	0	0	1	0	0	1	0	**12**	**21.8**
Influenza A	7	1	0	0	0	1	0	0	**9**	**16.4**
Human rhino/enterovirus	6	1	1	1	0	0	0	0	**9**	**16.4**
RSV	3	2	0	1	1	0	0	0	**7**	**12.7**
Coronavirus OC43	4	1	0	0	0	0	0	0	**5**	**9.1**
Coronavirus NL63	4	0	0	0	0	0	0	0	**4**	**7.3**
Coronavirus 229 E	3	0	0	0	0	0	0	0	**3**	**5.4**
Human *metapneumovirus*	3	0	0	0	0	0	0	0	**3**	**5.4**
Coronavirus HKU1	0	0	0	1	0	0	0	0	**1**	**1.8**
Parainfluenza	2	0	0	0	0	0	0	0	**2**	**3.6**
Total case	42	5	1	4	1	1	1	0	55	100
Infection rate per case group (%)	3.65	7.58	1.52	6.06	3.03	1.52	1.52	0.00	**3.79**	

*CVID* common variable immunodeficiency, *APDS* activated PI3 K-delta syndrome, *XLA* X-linked agammaglobulinemia, *HIES* hyper IgE syndrome, *NBS* Nijmegen breakage syndrome, *TTD* trichothiodystrophy

### Outcome data

There was no statistically significant difference in the within-group comparisons of the three measurements of ANC, ALC, IgG, IgA, and IgM levels (*p* > 0.05) (Supp. Table 1).

CVID patients received 39.3% (*n* = 24) IVIG, 19.6% (*n* = 12) cSCIG, and 13% (*n* = 8) fSCIG. The percentage of IRT use by diagnosis is presented in Supp Table 2.

Fifty-four patients (87%) experienced flu, six patients (6%) had sinusitis, two patients (2%) had pneumonia, and five patients (5%) had diarrhea. The diagnosis of infectious episodes was established through a comprehensive evaluation, which included a physical examination, standard sinus or chest X-ray, and assessment of elevated acute phase reactants. Systemic symptoms, such as headache, chills, and fever, were significantly more common in patients receiving IVIG, while local reactions were more frequently observed in patients receiving cSCIG and fSCIG (Supp. Table 3).

**Fig. 2:**
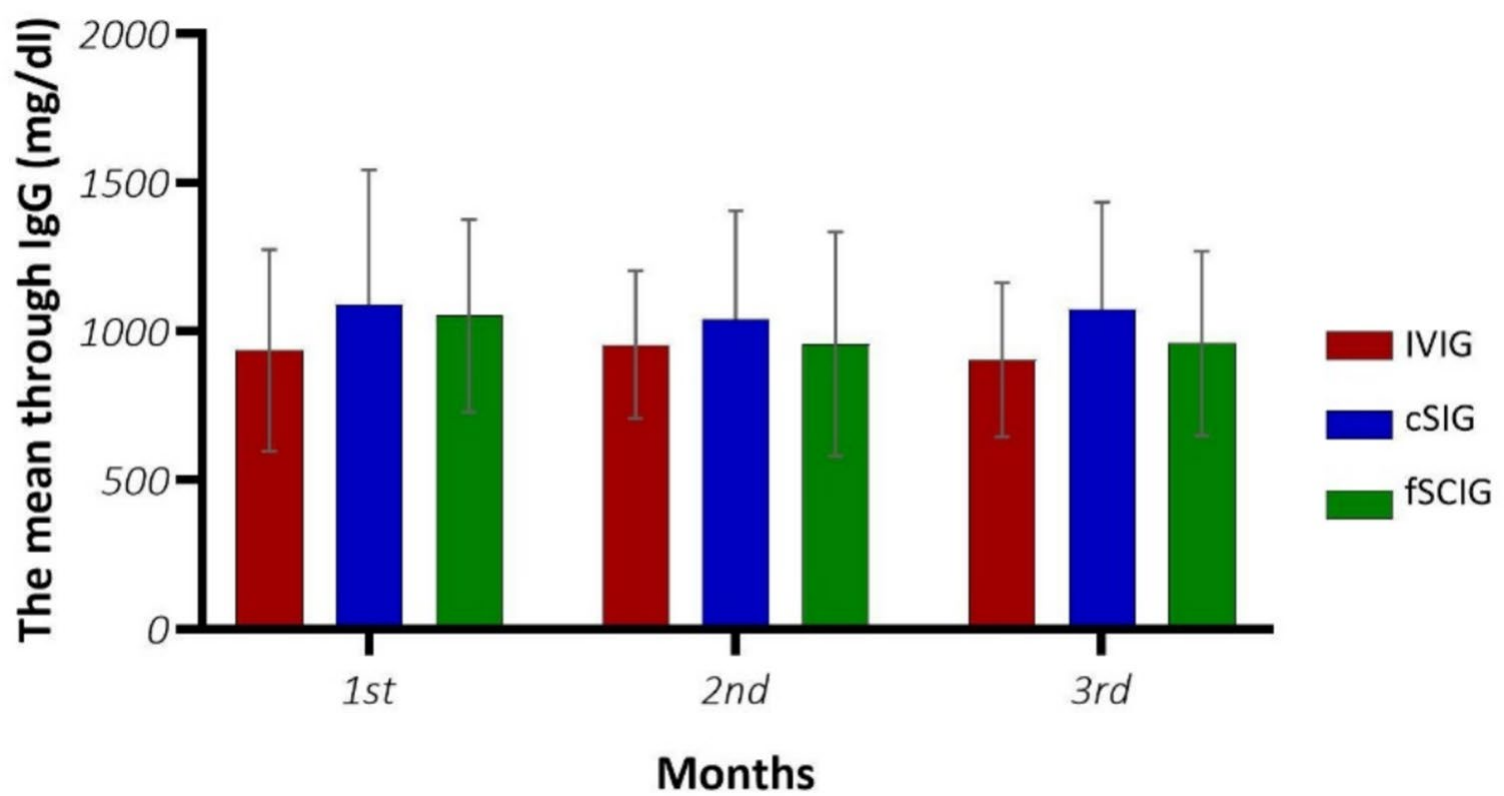
The three-month IgG trough levels by IRT administration route

When mean IgG levels were compared using repeated measures analysis according to months and infection status, no significant differences were found between groups, within groups, or in the group interaction (*p* > 0.05). Mean IgG levels were measured at the trough phase over 3 months across different IRT regimens (Fig. 2). According to the Ig treatment routes, the mean trough levels of IgG were 900 mg/dl for IVIG, 1200 mg/dl for fSCIG, and 1000 mg/dl for cSCIG. The mean monthly doses were 468.18 ± 132.25 mg/kg for IVIG, 462.92 ± 129.18 mg/kg for cSCIG, and 410.25 ± 65.32 mg/kg for fSCIG. There was no significant difference between the IgG trough levels and the 3-month IRT doses (*p* = 0.448).

## Discussion

IRT is a cornerstone in managing individuals diagnosed with IEI. Shared decision-making between patients and healthcare professionals is critical in determining the most appropriate administration route and product to minimize the risk of infections and achieve optimal treatment outcomes. This study found that immunocompromised patients receiving IRT via cSCIG had a lower incidence of viral infections than those receiving IVIG and fSCIG therapy. Consistent with our findings, Gupta and colleagues followed 51 patients who switched from IVIG to cSCIG over 12 months to assess infection frequency and IgG threshold levels [13]. The study demonstrated that cSCIG therapy reduced infection rates and serum IgG levels by 25%, which is higher than those previously observed with IVIG. While numerous studies comparing the effectiveness of different immunoglobulin products have primarily focused on the relationship between bacterial infections and the administered immunoglobulin dose, these studies typically involved small patient cohorts [14–16]. In our study, the incidence of viral infections was 50% lower with cSCIG compared to other routes of administration. The higher Ig trough values can explain this difference compared to fSCIG and IVIG. Infections mainly occurred when the IgG level reached the trough level. cSCIG requires more frequent infusion than IVIG but can be administered as a home-based treatment. When administering weekly cSCIG treatment, utilizing smaller doses at more frequent intervals maintains stable, higher trough serum concentrations. These concentrations remain constant between consecutive cSCIG infusions and are more effective in protecting against infections [17]. According to the research by Knutsen et al. [18], the group of patients receiving cSCIG demonstrated a statistically significant reduction in annualized infections of 0.8 ± 0.7 compared to the group receiving intravenous immunoglobulin therapy (IVIG) at 2.2 ± 1.2 per patient per year. The intravenous immunoglobulin (IVIG) volumes rapidly enter the systemic circulation, resulting in peak serum IgG levels within 24 h. Subsequently, these levels gradually decline over the treatment interval, causing fluctuating serum IgG levels. IgG’s half-life nears its end, and its ability to protect against infections diminishes gradually [19].

This study showed that adenovirus, influenza A, and human rhino/enterovirus were the most commonly detected agents. Other identified viruses, including RSV, were detected at a rate of 12.7%, while Coronavirus OC43 and Coronavirus NL63 were identified at rates of 9.1% and 7.3%, respectively. These data reflect the spectrum of virus infections seen during the winter period in immunocompromised patients. Although more than half of the patients (55.3%) experienced at least one of the specified infections, such as pneumonia or diarrhea, throughout the study, no patient required hospitalization, and two patients who experienced pneumonia were treated with oseltamivir. Thus, it is attributed to protecting IRT from severe and complex infection.

In our study, the mean IgA levels were 12.976 ± 38.924 mg/dl at 1 month, 1.001 ± 34.085 mg/dl at 2 months, and 11.97 ± 42.071 mg/dl at 3 months. A statistically significant correlation was found between infection rates and IgA levels (*p* = 0.016, 0.026, 0.018, respectively). IgA levels vary based on a patient’s genetic background and underlying conditions, but they are crucial for mucosal immunity. Monomeric serum IgA and secretory forms of IgA can neutralize and remove pathogens through various mechanisms. Human nasal IgA has greater neutralizing potency against influenza A viruses [20]. Commercial Ig preparations contain low IgA content, so their relative contribution to mucosal immunity is relatively limited. Immunoglobulin G (IgG) neutralizes viruses and activates immune responses. Notably, in patients with panhypogammaglobulinemia, IgG assumes a role in supporting mucosal immunity [21]. IgG is usually found at lower concentrations in healthy mucosal tissue. However, during periods of inflammation or when there is increased permeability of the epithelium, IgG can seep into mucosal areas and aid in immune defense. Recent research has indicated that the neonatal Fc receptor (FcRn) can actively move IgG across epithelial barriers, enabling it to access mucosal surfaces and neutralize pathogens [22]. This process is especially critical in the lower respiratory tract, where IgG is found in more significant amounts than IgA [23]. Therefore, although IgG is not the primary defense mechanism at mucosal sites, it serves a vital supplementary role, particularly in inflamed or damaged mucosal tissues.

The role of humoral immunity, as well as the innate and cellular immune response, in combating viral infections has been proven by the effectiveness of vaccines in preventing viral diseases. B cells have a pivotal role in the immune response, as they are responsible for both the production of antibodies and the modulation of the immune response through cytokine production. Additionally, they act as antigen-presenting cells (APCs) to T cells via MHC class II molecules. Therefore, B cells are essential in antiviral responses by several pathways [24]. In CVID, or X-linked agammaglobulinemia (XLA), B-cell dysfunction is associated with recurrent and persistent viral infections. Besides, genetic defects in some molecules that interact with T cells (such as NFKB1, NFKB2, and TNFRSF13B) in patients diagnosed with CVID can also lead to critical viral diseases. The largest denominator in our study group was patients diagnosed with CVID, and 7.87% had viral infections, the most common of which were adenovirus and influenza A.

Additionally, patients with NBS and AT with combined immunodeficiency showed high viral infections. Viral infections can cause lymphopenia, a decrease in the total number of circulating lymphocytes, through various mechanisms such as apoptosis, pyroptosis, and autophagy [25]. Triggering of viral infection may occur in some patients with pre-existing lymphopenia due to immunodeficiency. The study determined a statistically significant relationship between ALC and infections (Supp Table 1).

The total viral infection frequency was 3.79% for 3 months. In our study, the infection frequencies for IVIG, fSCIG, and cSCIG were 4.2%, 4.4%, and 2.5%, respectively. The infection rate was relatively high as we conducted this study in winter and over 3 months. The FIGARO study followed 128 patients to provide efficacy and tolerability of fSCIG for PID or SID for 36 months. The overall infection rate was 0% and 9.1% for patients during follow-up. This study confirms reasonable infection control and the feasibility of fSCIG in PID and SID patients at home and healthcare facilities [26]. A recent study found that the rates of confirmed acute bacterial infections in 30 patients with PID were similarly low among the three treatment modalities. According to the findings of this study, the annual incidence rates of upper respiratory tract infections, which encompassed streptococcal pharyngitis and viral respiratory tract infections, were reported as 4.17 for IVIG, 3.68 for SCIG, and 2.42 for fSCIG [10]. In a prospective study conducted on 44 adult patients diagnosed with primary antibody deficiency, viral agents were reported in 30.6% of the cases, bacterial agents were reported in 32.1%, and both viral and bacterial agents were detected in 12.5% [27].

Systemic reactions reported receiving IVIG were mild or moderate. Local skin reactions occurred in 18% (*n* = 2) with cSCIG and 9% (*n* = 1) with fSCIG. After changing the subcutaneous injection sites, the symptoms improved. Our study is the first to comprehensively compare viral infection rates in many immunocompromised patients. We have some limitations due to the small number of patients within specific subgroups.

In conclusion, this study suggests that cSCIG treatment is a more effective treatment option for immunocompromised patients prone to viral infection. Still, it is also a safe method to administer due to the optimum IgG threshold level and quality of life score it provides.

## Supplementary Information

Below is the link to the electronic supplementary material.

ESM 1 (PNG 35.4 KB)

ESM 2 (DOCX 22.0 KB)

## Data Availability

No datasets were generated or analysed during the current study.
