# Supplementary figures and images for: Comparison of the frequency of viral infections in patients with inborn errors of immunity receiving immunoglobulin by different routes
Source: Eur J Pediatr. 2025 May 30;184(6):373. doi: 10.1007/s00431-025-06201-w (PMC12122631; doi:10.1007/s00431-025-06201-w)

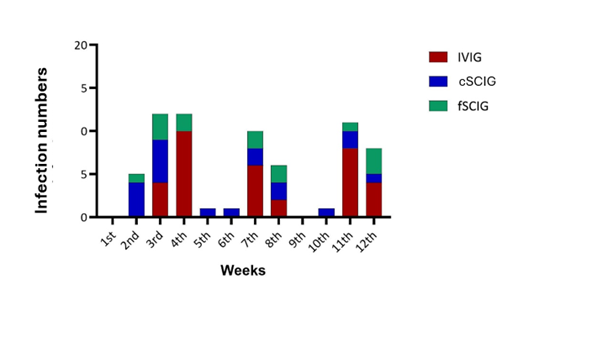

Supplement: Supplementary file 1 — (PNG 35.4 KB) [file 431_2025_6201_MOESM1_ESM.png]
