# Supplementary material for: Comparison of the frequency of viral infections in patients with inborn errors of immunity receiving immunoglobulin by different routes
Source: Eur J Pediatr. 2025 May 30;184(6):373. doi: 10.1007/s00431-025-06201-w (PMC12122631; doi:10.1007/s00431-025-06201-w)
Supplement: Supplementary file 2 — (DOCX 22.0 KB) [file 431_2025_6201_MOESM2_ESM.docx]

**SuppTable 1: Comparison of ANS, ALS, IgG, IgA, and IgM levels with infection status.**

|  |  | **Infection** | | | | | |
| --- | --- | --- | --- | --- | --- | --- | --- |
|  |  | No (n=22) | | Yes (n=39) | |  |  |
|  |  | Median / Mean | IQR/SD | Median / Mean | IQR /SD | Test statistic | p |
| **First month** | ANC | 2979,794 | 2057,728 | 3470,667 | 3052,529 | 2979,794 | 0.422 |
|  | ALC | 3145,987 | 2345,662 | 1902,431 | 1493,467 | 3145,987 | **0.034** |
|  | IgG | 1041,13 | 417,238 | 981,56 | 349,718 | 1041,13 | 0.581 |
|  | IgA | **12,976** | **38,924** | 70,202 | 111,172 | 12,976 | **0.016** |
|  | IgM | 33,906 | 82,203 | 40,94 | 71,905 | 33,906 | 0.882 |
| **Second month** | ANC | 3018,675 | 2176,535 | 3605,902 | 3142,551 | 3018,675 | 0.615 |
|  | ALC | 2877,394 | 2595,212 | 1827,268 | 1895,944 | 2877,394 | 0.078 |
|  | IgG | 957,275 | 286,617 | 996,951 | 322,624 | 957,275 | 0.632 |
|  | **IgA** | **1,001** | **34,085** | 60,084 | 99,166 | 1,001 | **0.026** |
|  | IgM | 38,098 | 88,195 | 36,05 | 57,912 | 38,098 | 0.769 |
| **Third month** | ANC | 3482,503 | 1718,371 | 4032,5 | 2048,051 | 3482,503 | 0.302 |
|  | ALC | 3455,823 | 2366,857 | 2400,455 | 1474,997 | 3455,823 | **0.037** |
|  | IgG | 941,974 | 305,135 | 976,979 | 305,926 | 941,974 | 0.713 |
|  | IgA | **11,97** | **42,071** | 70,08 | 111,82 | 11,97 | **0.018** |
|  | IgM | 51,105 | 61,164 | 43,072 | 68,143 | 51,105 | 0.918 |

IQR: interquartile range, SD: Standard deviation, ALC. Absolute lymphocyte count, ANC: Absolute neutrophil count

**Supp Table 2: The distribution of the IRT according to diagnoses**.

| **Diagnoses** | **IVIG (n, %)** | **cSCIG(n,%)** | **fSCIG(n/%)** |
| --- | --- | --- | --- |
| XLA | 2(2,27) | 0 | 0 |
| APDS | 2(3,27) | 0 | 0 |
| AT | 2(3,27) | 0 | 0 |
| CVID | 24(39,3) | 12(19,6) | 8(13) |
| HIES | 0 | 1(1,6) | 0 |
| HIM | 0 | 1(1,6) | 1(1,6) |
| NBS | 1(1,6) | 1(1,6) | 0 |
| SCID | 0 | 1(1,6) | 0 |
| TTD | 1(1,6) | 0 | 0 |
| **Total** | **32** | **16** | 10 |

Abbreviations: CVID, Common variable immunodeficiency APDS, Activated PI3K-delta syndrome XLA, X-linked agammaglobulinemia, HIES, Hyper IgE syndrome NBS, Nijmegen Breakage Syndrome TTD, Trichothiodystrophy

**Supp Table 3: Side effect distribution according to IRT routes**

|  | | **IRT route** | | | | | | |
| --- | --- | --- | --- | --- | --- | --- | --- | --- |
|  |  | **IVIG** | | **cSCIG** | | **fSCIG** | |  |
|  |  | n | % | n | % | n | % | P |
| **Headache** | Yes | 9 | 28 | 3 | 16.7 | 0 | 0.0 | **0.050** |
| **Nausea** | Yes | 5 | 15 | 2 | 11.1 | 0 | 0.0 | 0.069 |
| **Myalgia** | Yes | 5 | 9.4 | 1 | 5.6 | 1 | 9 | 0.622 |
| **Fever** | Yes | 8 | 25 | 2 | 11.1 | 0 | 0.0 | **0.042** |
| **Chills** | Yes | 8 | 25 | 1 | 5.6 | 0 | 0.0 | **0.044** |
| **Local reactions** | Yes | 2 | 7,5 | 2 | 18 | 1 | 9 | **0.048** |
| **Aseptic meningitis** | Yes | 1 | 0.0 | 0 | 0 | 0 | 0 | 0.222 |
